# Highly-Sensitive Detection of Volatile Organic Compound Vapors by Electrospun PANI/P3TI/PMMA Fibers

**DOI:** 10.3390/polym12020455

**Published:** 2020-02-16

**Authors:** Duy Linh Vu, Tz-Feng Lin, Ting-Han Lin, Ming-Chung Wu

**Affiliations:** 1 Department of Chemical and Materials Engineering, Chang Gung University, Taoyuan 33302, Taiwan; vuduylinhbk@gmail.com (D.L.V.); cgu.tinghanlin@gmail.com (T.-H.L.); 2 Department of Fiber and Composite Materials, Feng Chia University, Taichung 40724, Taiwan; tflin@fcu.edu.tw; 3 Green Technology Research Center, Chang Gung University, Taoyuan 33302, Taiwan; 4 Division of Pediatric Neonatology, Department of Pediatrics, Chang Gung Memorial Hospital, Linkou, Taoyuan 33305, Taiwan

**Keywords:** P3TI, freestanding fibers, VOCs detection, electrospinning, UV/ozone treatment

## Abstract

Detection of volatile organic compounds (VOCs) is one of the essential concerns for human health protection and environmental monitoring. In this study, the blending fibers using a donor-acceptor copolymer were fabricated by electrospinning technique and subsequent UV/ozone treatment. The donor-acceptor polymers were polyaniline, P3TI, and poly(methyl methacrylate) (PANI/P3TI/PMMA) fibers with a cylindrical structure and uniform morphology. VOCs were directly adsorbed by the copolymer materials assembled onto a glass surface or metal framework scaffold. Under optimal conditions, the PANI/P3TI/PMMA fibers exhibit rapid response and high selectivity to VOC vapors within 30 min of UV/ozone treatment. Additionally, the optical transmittance changes of the freestanding fibers show significant improvement of more than 10 times to those fibers on glass substrates. It is speculated that the presence of P3TI leads to the formation of a heterojunction and increases the electron reception behavior. The modification of the electronic structure as exposed to VOC vapors tend to significantly alter the optical absorbance of the fibers, leading to the excellent sensing at low VOC concentration.

## 1. Introduction

To detect environmentally hazardous gases, many modern technologies have been developed to avoid and control the concentration of harmful gases. Numerous volatile organic compounds (VOCs) are known: various kinds of organic chemical gases and ingredients in a large number of industrial products, such as fuel, paints, cosmetics, composite wood, and even some ordinary exhaust fumes, like cooking, smoking, and wood-burning. With the VOC feature of simple evaporation at room temperature, human health can easily be affected through breathing or skin contact [1,2,3]. Therefore, sensing VOC vapors at low concentration is extremely important and necessary for health protection [4,5]. Based on a cross-reactive sensor of conductivity, optical response, and volume, when exposed to different types of gases, the sensitivity can be precisely identified by changes in optical transmittance [6,7].

Numerous sensing devices fabricated by conducting polymers have been investigated and applied in many fields. Conducting polymer has outstanding advantages when used in chemical and biological sensors with excellent selectivity and sensitivity [8]. It is also an attractive material for VOC detection because of the benefits of easy fabrication, detectable optical changes, high sensitivity, and low poisoning effect [9,10,11,12]. 

Polyaniline (PANI) has been widely used over the past 20 years for the detection of various gases, such as H_2_O, NH_3_, NO_x,_ and hydrogen, due to the redox reaction of a nitrogen atom in PANI [9,13,14,15]. The structure of PANI from a polymerization reaction of aniline includes two functional groups, a reduced [B–N–N–NH] repeat unit and an oxidized [B–N=Q=N^−^] repeat unit, where B indicates benzenoid, and Q indicates a quinoid ring. By the redox reaction, the benzenoid and quinoid ring can be interchanged with each other. When the ration of reduced units to oxidized units are 1:1 (i.e., benzenoid:quinoid = 3:1), PANI consisted of emeraldine salts and becomes electrically conductive. Through the reversible acid/base equilibrium, polyaniline can be changed from the emeraldine base with blue color and the emeraldine salt with green color [9,16]. However, some VOC sensors show low response due to the limited magnitude change of the electronic structure compared to NH_3_, NO_x_ gases and, hence, PANI is typically combined with other materials, such as PVA, PPy, ZnO, CuO, and Ag, for VOC sensing applications [14,16,17,18,19]. 

Conjugated polymers have been widely applied in chemical and biological sensors due to outstanding advantages, including excellent sensing response, and high selectivity and sensitivity [8]. P3TI was chosen as one of the promising donor (D)-acceptor (A) copolymers to increase optical responses and achieve high sensitivity or selectivity. D–A copolymer shows a combination of a high-lying HOMO energy level (highest occupied molecular orbital) and a low-lying LUMO energy level (lowest unoccupied molecular orbital) in their narrow optical bandgaps [20,21,22]. Additionally, P3TI shows an easy-to-synthesize and straightforward structure. There are some properties important for gas sensors to achieve high sensitivity and selectivities, like high air stability and hole mobility [23]. 

Using the electrospinning process can produce fibers with a majority of materials, for example, polymers, composites, semiconductors, and ceramics [24,25,26]. When considering the flexibility of the sensor manufacture, electrospinning is a state-of-the-art method to fabricate fibers with benefits including solution process, high specific surface area, ease of material combination, mass production, and relatively low start-up cost. In addition, the electrospinning technique can easily control the morphology of free-standing films, such as fiber diameter, shape, and thickness of the film. Poly(methyl methacrylate) (PMMA) is used as the matrix of the sensing material because of its transparent and good mechanical properties [27,28,29,30]. Therefore, we designed PANI/P3TI/PMMA fibers to improve the detection of VOCs.

In this study, PANI/P3TI/PMMA fibers were fabricated by the electrospinning technique to detect VOC vapors. The free-standing fibers were, respectively, deposited onto a glass substrate and metal framework before being treated with a UV/ozone system. Then, the response of sensing fibers was tested practically under various VOC vapors with different concentrations. The effects of UV/ozone treatment, loading substrates were discussed. The PANI/P3TI/PMMA fibers exhibited excellent sensitivity and low detection limits to VOCs, especially to butanol vapors.

## 2. Experiment Section

### 2.1. Preparation of PANI/P3TI/PMMA Fibers

Polyaniline emeraldine base (PANI, M.W. ~65,000) and poly(methyl methacrylate) (PMMA, M.W. ~996,000) were purchased from Sigma-Aldrich (Sigma-Aldrich, St. Louis, MO, USA). P3TI was fabricated according to a previous study [31]. At first, PANI and P3TI, in the ratio of 10:1, were dissolved in N-methyl-2-pyrrolidone (NMP, Sigma-Aldrich, St. Louis, MO, USA) with continuous stirring at 90 °C for 24 h. Then PMMA was added to the solution and continuously stirred at 60 °C until a homogeneous PANI/P3TI/PMMA solution was obtained. More detail can be found in the supporting information. The mixture solution was loaded into a 10 mL syringe and allowed to undergo an electrospinning process to fabricate the PAIN/P3TI/PMMA fibers. In the electrospinning process, to have a stable process, we need to balance the flow rate of the solution through the syringe and the flow rate of the solution at the tip under certain electric propulsion. The parameters of the system are set up at a constant value as below. The tip-to-collector distance is 10 cm; the speed of the collector is 600 rpm; the applied voltage is 20 kV; the flow rate of the PANI/P3TI/PMMA solution is 0.6 mL h^−1^ for 3 h. The ambient condition is 25 °C and 50% relative humidity. The fibers were harvested by directly assembling onto the glass surface or metal framework before treatment by a UV/ozone system.

### 2.2. Characterization

To study the surface morphology and analyze the contents of the elements therein, we observed specimens under field emission scanning electron microscope with energy-dispersive X-ray spectroscopy (FESEM, model SU8010, Hitachi High-Technologies Corp., Tokyo, Japan) equipped with EDS (XFlash Detector 5030, Bruker AXS, Karlsruhe, Germany). Fourier-transform infrared spectroscopy (FTIR, model Horiba FT-730, Minami-ku, Kyoto, Japan) was used to evaluate the functional groups and chemical bonding of PANI and P3TI. The FTIR spectra were recorded in the range of 4000 to 450 cm^−1^ with a resolution of 2.0 cm^−1^.

### 2.3. VOC Detection

VOC concentration was controlled by delivering a certain amount of organic chemical solvents, including n-butanol (99.5%, Acros, Fair Lawn, NJ, USA), dimethylformamide (DMF, 99.8%, Acros, Fair Lawn, NJ, USA), toluene (99.8%, Acros, Fair Lawn, NJ, USA), n-propanol (99.5%, Acros, Fair Lawn, NJ, USA), and chlorobenzene (CB, 99.8%, Acros, Fair Lawn, NJ, USA) into an 80 cm^3^ chamber (Figure S1) and waiting for its evaporation at room temperature in equilibrium. After the VOC solvent was volatilized, PANI/P3TI/PMMA fibers were placed in the chamber. The transmission spectra were studied at a working temperature of 25 °C by UV–VIS spectrophotometry (V-730, Jasco, Tokyo, Japan) with the range of the wavelength from 400 to 1100 nm and a resolution of 0.5 nm. To diminish the noise signal during VOC measurements, we investigated the influence of the absorption of the gas chamber and VOC vapor and the stability of the optical system in the blank experiment. Figure S2 shows the transmittance at 620 nm without sensing material in the chamber for 1800 s. Firstly, the chamber without VOC vapor was placed in the UV–VIS spectrometer to record the optical signal for 500 s. The transmittance was decreased from 100% to 87.63% due to the absorption of the quartz-made gas chamber. At time t = 501 s, n-butanol vapor with 2000 ppm was released into the chamber and we continued to record the transmittance signal until t = 1800 s. From the results of both processes, we determined that the noise signal of transmittance was about 0.0304% in the blank experiment. On the other hand, the response of the sensing process is defined by the extinction change, which converted from the transmission signal. The extinction at 620 nm is calculated by Equation (1) [30]:

(1) $$E_{t}=\log\left(\frac{100-T_{\mathrm{baseline}}+T_{t}}{100}\right)$$

where E_t_ is the extinction of the sensing material exposed to VOC vapors at time t, T_baseline_ is the transmittance of the empty gas chamber, and T_t_ is the transmittance at exposure time t.

Afterward, the extinction change (ΔE) at exposure time t is expressed as follows:

(2) $$\Delta E_{t}=E_{i}-E_{t}$$

where E_i_ is the initial extinction before exposure to VOC vapors, and E_t_ is the extinction of exposure time t. Since the extinction change of the sensing noise signal is about 0.001, it is used as a reference to screen the noise. Namely, the response of sensing material exposed to VOC vapors is recognized as its extinction change is greater than 0.001.

## 3. Results and Discussion

**Figure 1:**
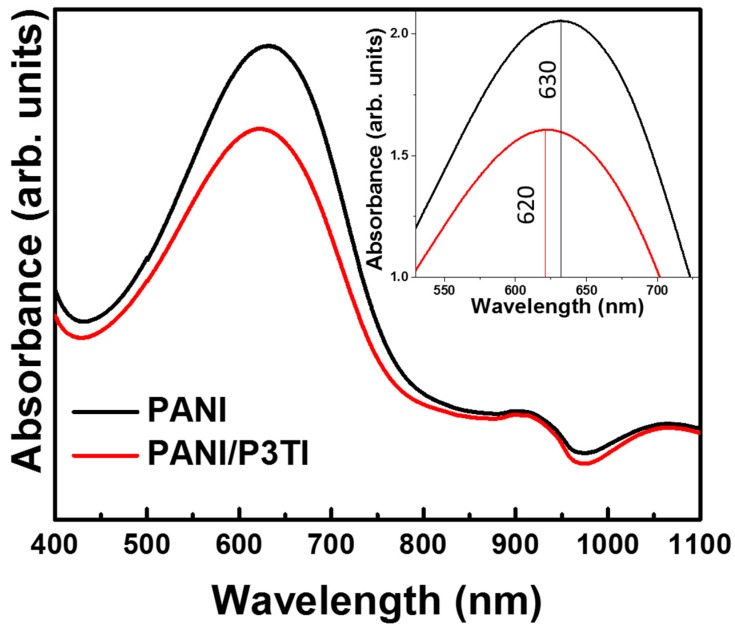
Absorbance spectra of PANI and PANI/P3TI.

To determine the transition of the excited state, we used UV–VIS spectroscopy to examine the absorption spectra of PANI and PANI/P3TI, as shown in Figure 1. The absorption spectra of PANI show the highest peak at 630 nm of the optical light source, which represents the blue color of the PANI emeraldine base. After P3TI was doped into the solution, the highest peak shifted slightly to a lower wavelength at 620 nm. It can be seen that the optical properties of the sensing material was almost unchanged when doped with P3TI. Thus, the absorbance at a wavelength of 620 nm was used for VOC detection.

**Figure 2:**
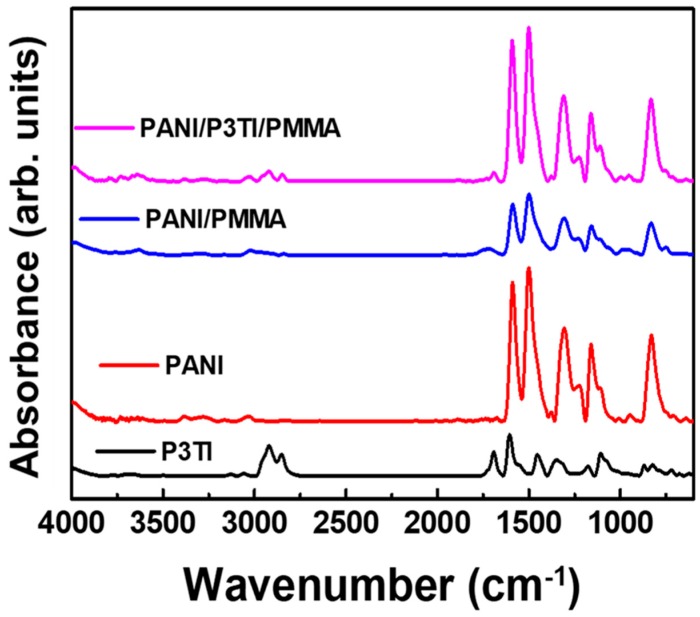
FTIR spectra of PANI/PMMA, PANI/P3TI/PMMA, pristine PANI, and P3TI.

Figure 2 represents the FTIR spectroscopic analysis for the PANI/PMMA, PANI/P3TI/PMMA fibers, and the pristine PANI, P3TI. Compared with the main absorption peaks of the pristine PANI, both PANI/PMMA and PANI/P3TI/PMMA fibers have the same peak located at 1587, 1495, 1302, 1163 cm^−1^, corresponding to the quinoid ring (N=Q=N), N–H stretching, C–N stretching, C=C benzenoid ring, and quinoid ring stretching [32]. Considering the spectrum of pristine P3TI, we observed that two characteristic peaks of P3IT at 1693 and 708 cm^−1^ could be recognized. The presence of C=O and C–S stretching is indicated [33]. Although the peak was not apparent in the spectrum of PANI/P3TI/PMMA, the low ratio of P3TI in the PANI/PMMA composite was considered. The FTIR spectra results confirmed that P3TI was successfully incorporated with the polymer matrix and maintained in its original chemical bonding after the blending and electrospinning process.

**Figure 3:**
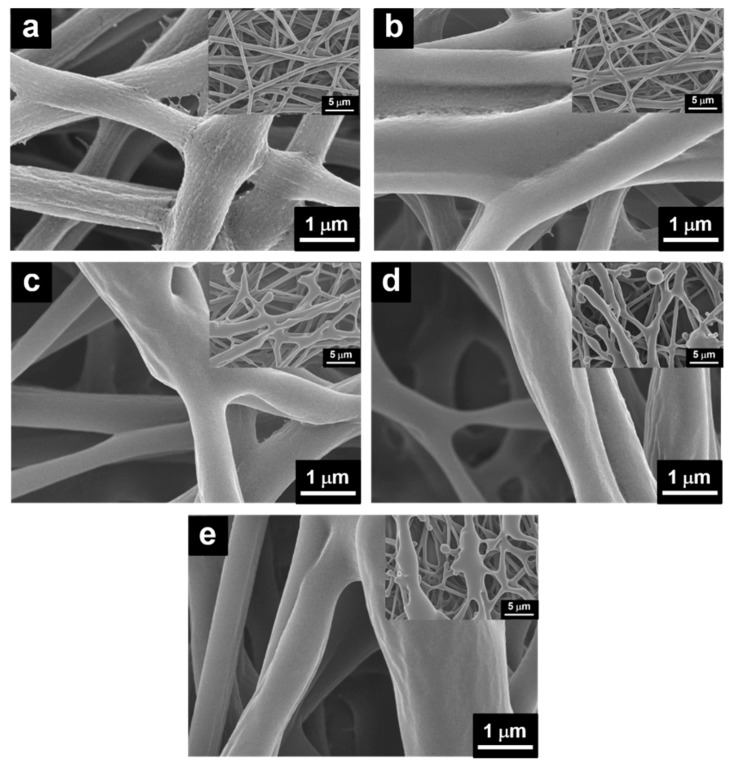
SEM images of PANI/P3TI/PMMA fibers treated by UV/ozone at different times, (**a**) 0 min, (**b**) 10 min, (**c**) 20 min, (**d**) 30 min, and (**e**) 40 min.

Figure 3 shows the morphologies of the PANI/P3TI/PMMA fibers with various UV/ozone treatment times. PANI/P3TI/PMMA fibers showed a cylindrical form, highly uniformity and continuous, as shown in Figure 3a and Figure S3. The related average diameter of the fibers is about 500 nm. The rough surface of the blending fibers was observed owing to the manufacturing process of the electrospinning system [30]. However, with the UV/ozone treatment, the surface of the fibers become smooth (Figure 3b–e). Additionally, the morphology of fibers converted to ribbon form, leading to an increase in the diameter with the increase of treatment time. Notably, when the treatment time is higher than 20 min, the morphology of fibers was changed significantly. Some fibers were broken, and others appeared interconnected with adjacent fibers. This was attributed to oxidation by ozone. The hydroxyl groups (–OH) were produced and reduced some of the functional groups, such as C–O, O–C=O, leading to fiber structure change [31,33]. According to the results, the sensitivity of the fibers can be enhanced.

**Figure 4:**
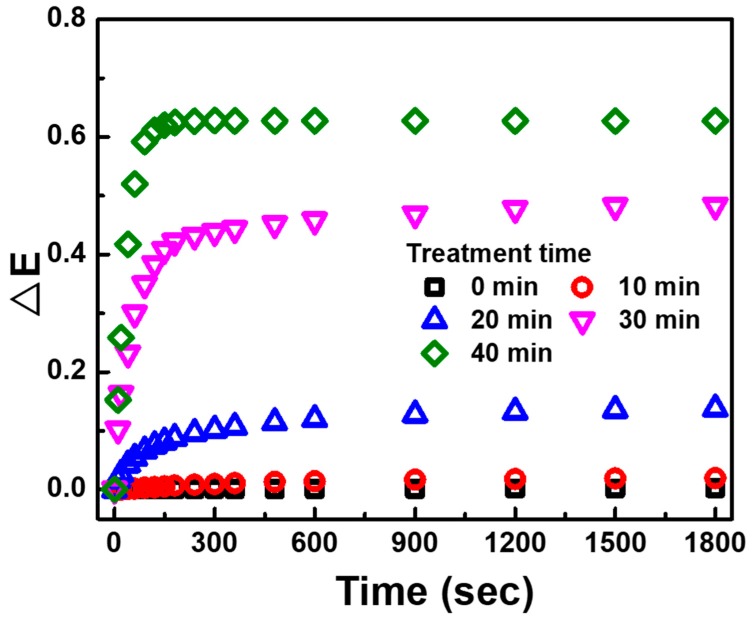
Extinction change of PANI/P3TI/PMMA fibers exposed to 2000 ppm n-butanol with various UV/ozone treatment time.

To improve VOC sensing performance, we studied the various UV/ozone treatment times, which is the critical parameter to change the response in VOC sensing. Figure 4 shows the extinction change of PANI/P3TI/PMMA fibers exposed to 2000 ppm n-butanol for 1800 s with various UV/ozone treatment times ranging from 0 to 40 min. Due to the low surface energy of thermoplastics, and the derived hydrophobicity or weak hydrophilicity [34], the bonding strength between PANI/P3TI/PMMA fibers and VOC molecules is limited and tends toward low sensitivity. It is noted that the response of the sensing fibers without treatment and with 10 min treatment was nearly unchanged when exposed to 2000 ppm n-butanol vapor for 1800 s. Afterward, with the UV/ozone treatment for more than 20 min, the ester group on PMMA quickly becomes oxidized with ozone, and then produced hydroxyl groups (–OH). These hydroxyl groups create hydrogen or covalent bonds on the surface fibers, suggesting that the surface energy of PANI/P3TI/PMMA fibers would be increased. Therefore, the extinction change of the fibers dramatically increased when the treatment time was prolonged from 20 to 40 min. However, PANI/P3TI/PMMA fibers were unstable in high relative humidity conditions as the treatment time was up to 40 min, as shown in Figure S4. According to the result, the acceptable treatment times are 20 and 30 min due to the stable and effective response to n-butanol.

**Figure 5:**
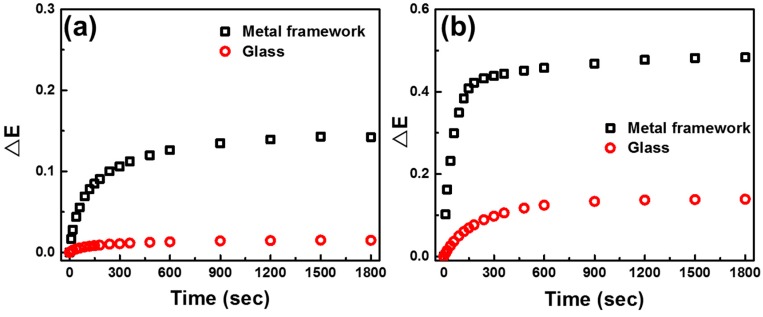
Extinction change of PANI/P3TI/PMMA fibers deposited on different substrates exposed to 2000 ppm n-butanol with (**a**) 20, and (**b**) 30 min of UV/ozone treatment.

We have successfully harvested the PANI/P3TI/PMMA fibers with a metal framework. The extinction change at 620 nm of PANI/P3TI/PMMA fibers with different substrates, when exposed to 2000 ppm n-butanol, is shown in Figure 5. The PANI/P3TI/PMMA fibers deposited on the glass substrates were only exposed to VOCs with a single side, while those loaded on the metal framework substrates provided double faces exposed to VOCs. Moreover, during the surface treatment, the fibers can be comprehensively bared to ozone due to the double-side exposure as loaded on the metal framework. This would boost the efficiency of the surface treatment. Compared to glass substrates, the response of PANI/P3TI/PMMA fibers on metal framework substrates was significantly magnified about nine times with 20 min UV/ozone treatment (Figure 5a), and three times after 30 min UV/ozone treatment (Figure 5b), respectively. Thus, 30 min of UV/ozone treatment time was chosen for further study.

**Figure 6:**
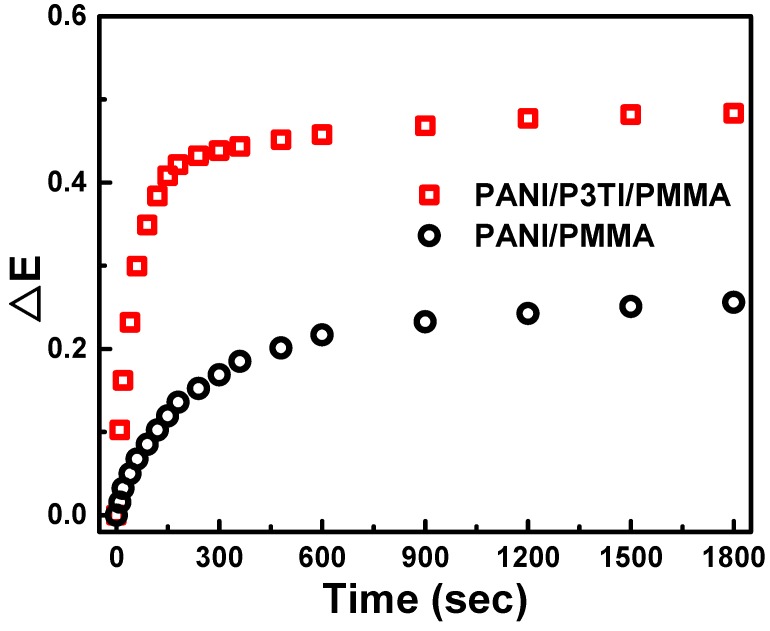
Extinction change of PANI/PMMA and PANI/P3TI/PMMA fibers when exposed to 2000 ppm n-butanol.

Based on the spectral changes of absorption and fluorescence shifts when the polymer backbone reacts with particular ions, the sensitivity of the chemical sensor will be recognized [8,35]. P3TI-doped PANI/PMMA is an extremely rational method for promoting the response of VOC sensing. Figure 6 shows the extinction change of PANI/PMMA and PANI/P3TI/PMMA fibers exposed to 2000 ppm n-butanol. The maximum extinction change grew from 0.256 to 0.483, an improvement around 1.9 times after P3TI was incorporated into PANI/PMMA. Additionally, the response time of PANI/P3TI/PMMA fibers was relatively short, compared to PANI/PMMA fibers. This result indicates that P3TI significantly enhanced the sensitivity and shortened the response time for VOC sensing measurements.

**Figure 7:**
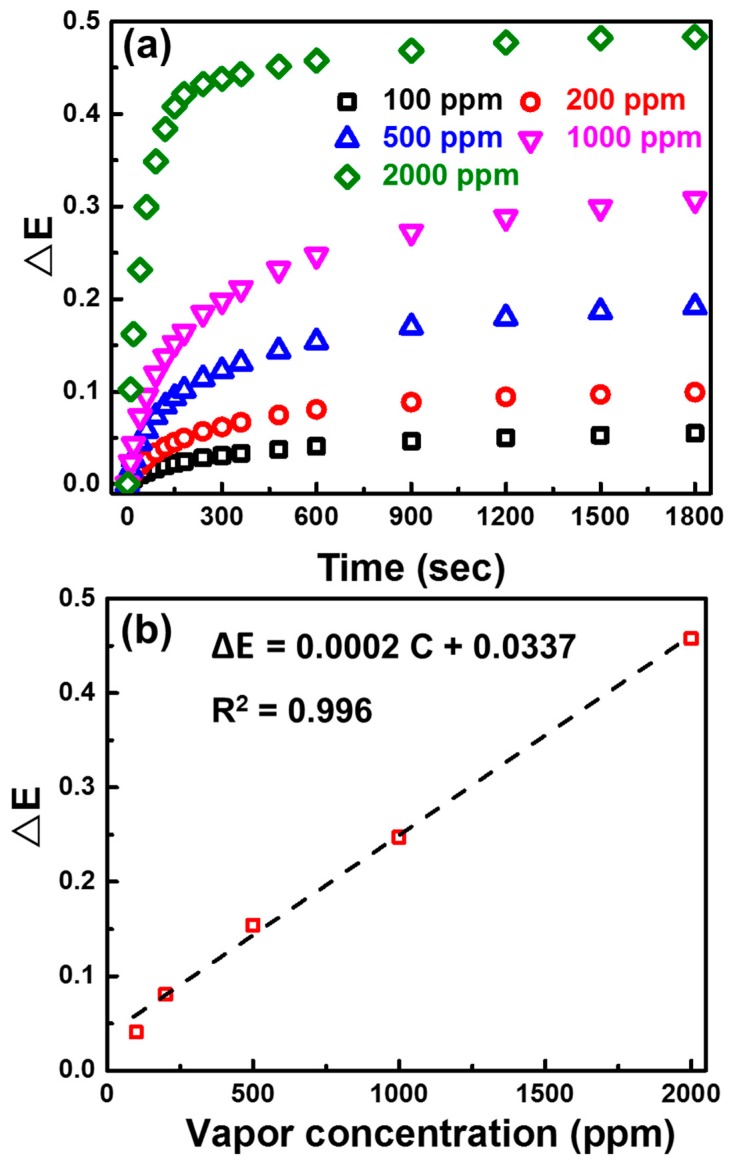
(**a**) Extinction change of PANI/P3TI/PMMA fibers, when exposed to several n-butanol concentrations, and (**b**) the calibration curves of PANI/P3TI/PMMA fibers after 600 s exposed to n-butanol vapor.

The selective responses of various n-butanol concentrations of PANI/P3TI/PMMA fibers are examined and presented in Figure 7a. The extinction change depends on the n-butanol concentrations. The detection limit of the sensing fibers was investigated through VOC sensing measurements (Figure S2) with a concern to background noises. When the extinction change of PANI/P3TI/PMMA fibers is greater than the sensing noise signal (∆E = 0.001), the response of VOCs sensing will be identified. Hence, the detection limit of the PANI/P3TI/PMMA fibers for n-butanol is estimated at 100 ppm. As exposure to specific concentrations lowered to 100 ppm, it was difficult to show any responses of VOC sensing. Herein, we believed that the detection role is related to VOCs adsorption and condensation onto the blending fibers, as well as the solubility. As treated by UV/ozone, hydroxyl groups might be induced on the surface of the PMMA fiber. These conjugated polymers might also be modified and activated, changing their excited state, and becoming more sensitive. 1-Butanol was not only attracted due to hydrogen bonding and polar induced dipole bonding, but also adsorbed depending on the boiling point. When excess VOCs were loaded on the blending fibers, swelling occurred, and fibers collapsed. Interestingly, the solution parameters of 1-butanol and the PMMA matrix are close, contributing to the extinction change significantly [36].

The relationship between VOC vapor concentration and extinction change was discovered. We demonstrated the calibration curve of ∆E toward the concentration of VOC vapors, fitted by the experimental data, and shown as follows: ∆E = k⋅C + A, where k is the sensitivity coefficient, C is the concentration of VOC vapors, and A is a constant [37]. In Figure 7b, the calibration curves for n-butanol vapor is ∆E = 0.0002C + 0.0337.

**Figure 8:**
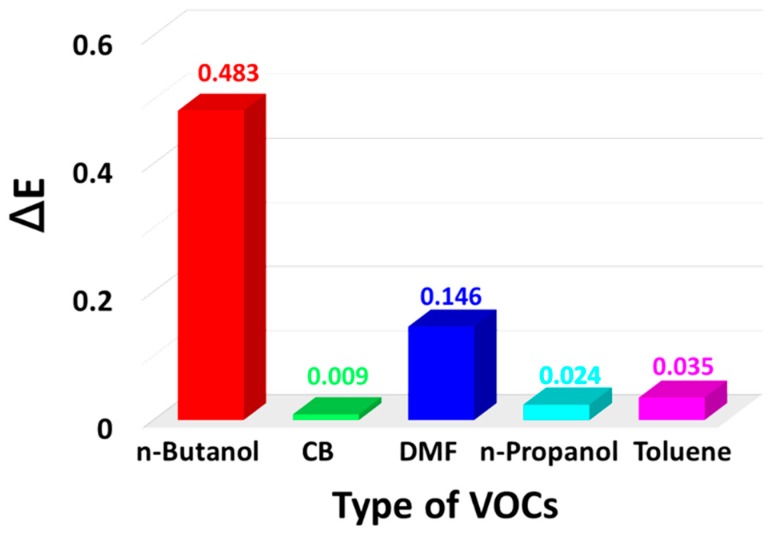
The maximum extinction change of PANI/P3TI/PMMA fibers exposed to 2000 ppm VOC vapors.

To evaluate the selectivity of PANI/P3TI/PMMA fibers, we reported the response of various VOC detections. Figure 8 shows the maximum extinction change of PANI/P3TI/PMMA fibers after 1800 s exposed to 2000 ppm VOC vapors. The extinction changes are 0.483, 0.009, 0.146, 0.024, and 0.035 for n-butanol, CB, DMF, n-propanol, and toluene, respectively. It can be observed that the sensing fibers show a good response to n-butanol and DMF vapors. This is attributed to the formation of hydrogen bonding on the surface and the outstanding solubility of the conjugated polymer in DMF [36]. The extinction changes of the above VOC vapors are many times higher than the sensing noise signal (∆E = 0.001). Therefore, the PANI/P3TI/PMMA fibers show high selectivity in different VOC sensing applications.

**Figure 9:**
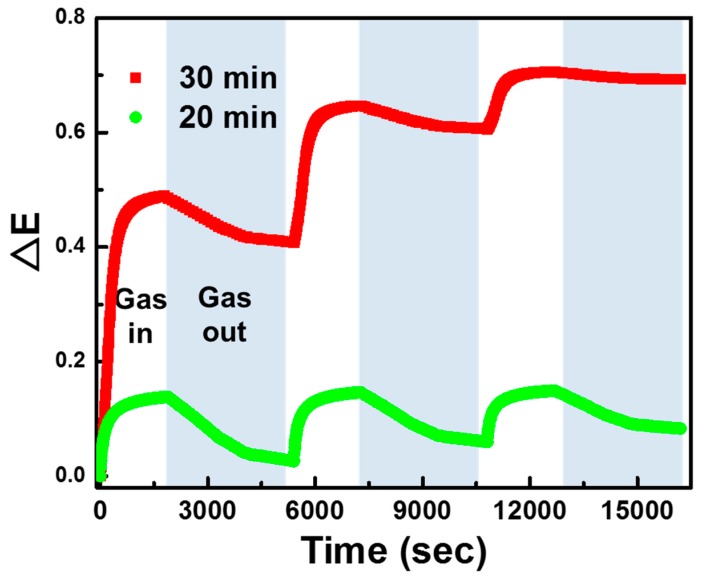
Reversibility of PANI/P3TI/PMMA fibers with 20 min and 30 min of UV/ozone treatment as exposed to 2000 ppm n-butanol.

We also investigated the reversibility of PANI/P3TI/PMMA fibers. The response–recovery cycles of the sensing fiber were recorded three times at the same condition, exposed to 2000 ppm n-butanol, as shown in Figure 9. After the sensing material inserted into the chamber, the response of PANI/P3TI/PMMA fibers toward n-butanol vapor was very rapid, and the response times of sensing fibers with both 20 min and 30 min UV/ozone treatment were less than 10 s. However, PANI/P3TI/PMMA fibers with UV/ozone treatment for 30 min cannot recover to the initial state after long-time exposure to n-butanol. It is deduced that the sensing fibers were eclipsed by VOC vapor and deformed dramatically. Then, the initial extinction of every cycle would be altered, and the extinction change is limited. The extinction change after three cycles reduced from 0.483 of the first cycle to 0.099 of the third cycle. Interestingly, PANI/P3TI/PMMA fibers with UV/ozone treatment for 20 min show better reversibility. The extinction change of the sensing fiber almost recovered to the initial level. Nevertheless, the stability of the response was still limited, reduced from 0.140 to 0.079, and showed a low response. In summary, the UV/ozone treatment for PANI/P3TI/PMMA fibers reduces the reversibility but significantly increases the VOC response. It is indicated that the reversibility of PANI/P3TI/PMMA fibers can only be acceptable with fewer cycles, but the ultrasensitive response to VOCs is appropriate for the application of a disposable sensor.

## 4. Conclusions

Highly-sensitive PANI/P3TI/PMMA fibers for the detection of VOC vapors at a working temperature of 25 °C were successfully fabricated by the electrospinning technique, and were uniform, continuous, and well-dispersed. Various UV/ozone treatment times for the sensing fibers were investigated. At a treatment time of 30 min, the surface energy of the sensing fibers could be improved and the sensing fibers become active, leading to a high response. Moreover, the freestanding PANI/P3TI/PMMA sensing fibers were collected onto a metal framework, providing a larger reactive surface. Compared to the collection onto a glass substrate, the response can be boosted by three times. The PANI/P3TI/PMMA fibers exhibited excellent sensitivity, low detection limit, and response to multiple VOC vapors, especially to n-butanol vapors. The detection limit for n-butanol is about 100 ppm, and the response time is less than 10 s.

## Supplementary Materials

The following are available online at https://www.mdpi.com/2073-4360/12/2/455/s1, Figure S1: Illustration of the chamber of VOCs detection for UV-vis spectra measurement. Figure S2: The transmittance signal at 620 nm in the blank for 1800 s. Figure S3: (a) FESEM image and (b–e) EDS elemental mappings of the PANI/P3TI/PMMA fiber, including (b) carbon, (c) nitrogen, (d) sulfur, and (e) oxygen. Figure S4: Extinction change at 620 nm of PANI/P3TI/PMMA fibers with various UV/ozone treatment time when exposed to 75% relative humidity.
