# Supplementary material for: Highly-Sensitive Detection of Volatile Organic Compound Vapors by Electrospun PANI/P3TI/PMMA Fibers
Source: Polymers (Basel). 2020 Feb 16;12(2):455. doi: 10.3390/polym12020455 (PMC7077691; doi:10.3390/polym12020455)
Supplement: Supplementary file 1 [file polymers-12-00455-s001.pdf]

Supporting Information

Highly-Sensitive Detection of Volatile Organic Compound Vapors by Electrospun PANI/P3TI/PMMA Fibers

Duy Linh Vu [1,†], Tz-Feng Lin [2,†], Ting-Han Lin [1,†] and Ming-Chung Wu [1,3,4,*]

[1] Department of Chemical and Materials Engineering, Chang Gung University, Taoyuan 33302, Taiwan; vuduylinhbk@gmail.com (D.L.V.); cgu.tinghanlin@gmail.com (T.-H.L.)
[2] Department of Fiber and Composite Materials, Feng Chia University, Taichung 40724, Taiwan; tflin@fcu.edu.tw
[3] Green Technology Research Center, Chang Gung University, Taoyuan 33302, Taiwan
[4] Division of Pediatric Neonatology, Department of Pediatrics, Chang Gung Memorial Hospital, Linkou, Taoyuan 33305, Taiwan
* Correspondence: mingchungwu@cgu.edu.tw
† These authors contributed equally to this work as first author.

Preparation of the electrospun precursor solution

The preparation process of the solution was similar to the reported of our previous study [30]. At first, 20 0 mg PANI and 2.0 mg P3TI were dissolved in 9.0 ml NMP, and then the mixture was stirred at 90°C for 24 h. Subsequently, 980 mg PMMA was added to the solution and continuous stirring at 60°C until the homogeneous PANI/P3TI/PMMA solution was obtained.

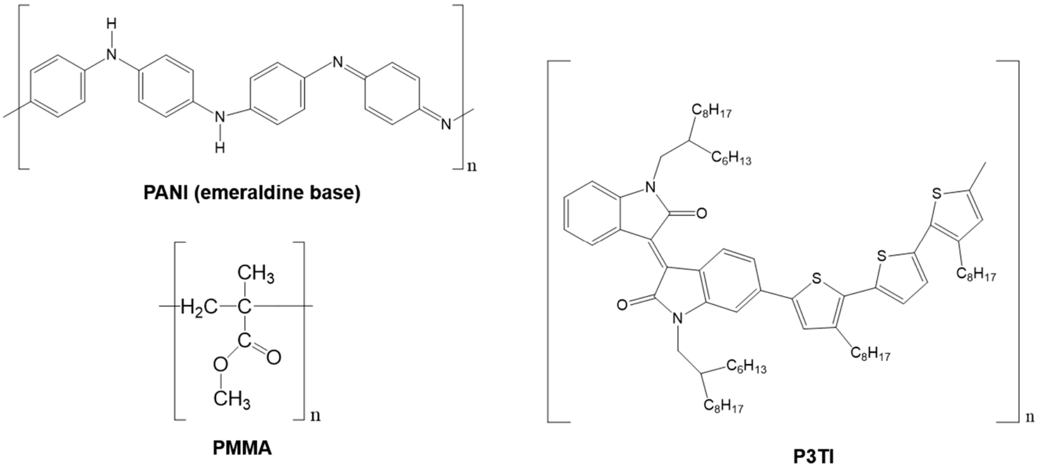

Scheme S1. The molecular structure of the PANI, P3TI, and PMMA.

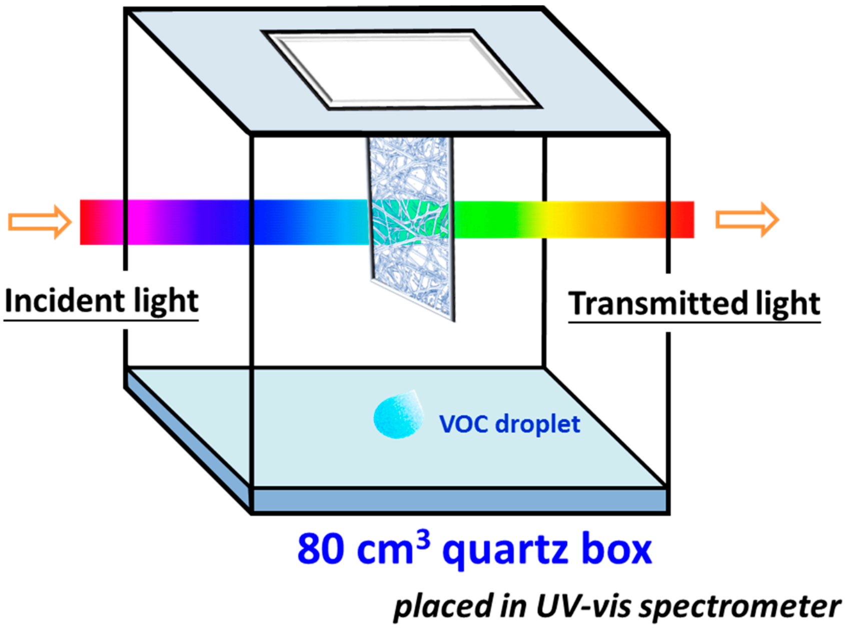

Figure S1. Illustration of the chamber of VOCs detection for UV-vis spectra measurement.

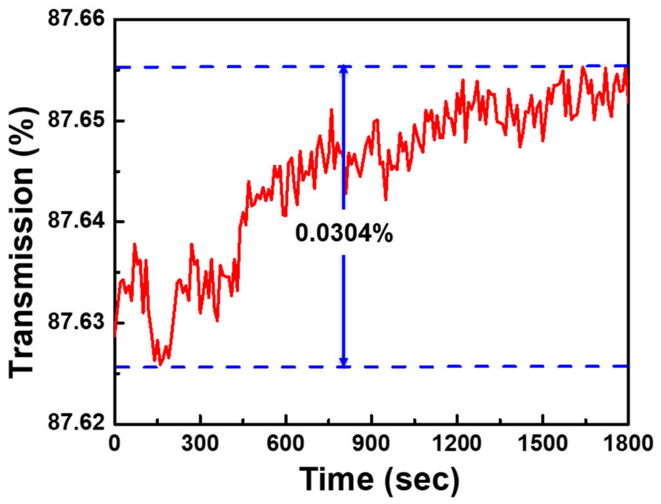

Figure S2. The transmittance signal at 620 nm in the blank for 1800 s.

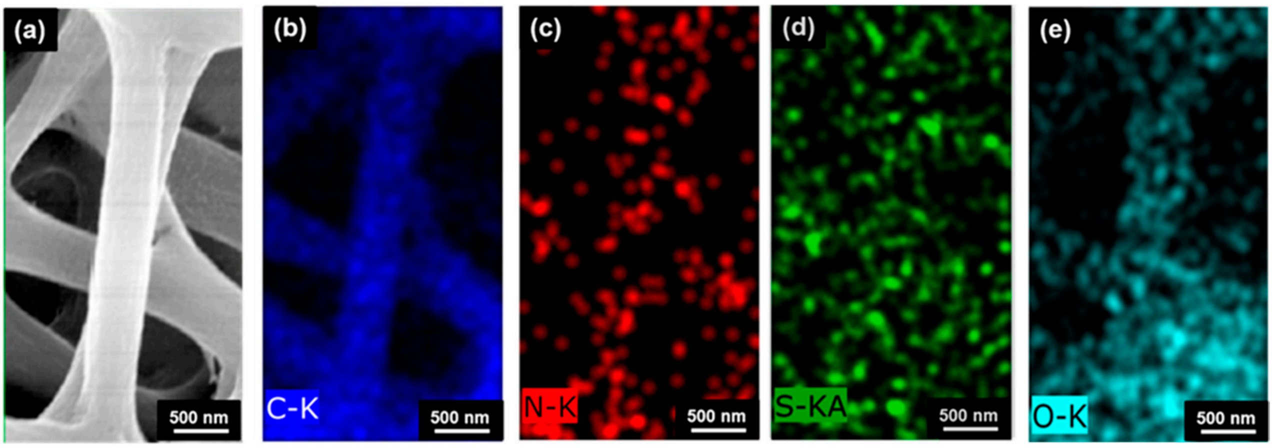

Figure S3. (**a**) FESEM image and (**b**–**e**) EDS elemental mappings of the PANI/P3TI/PMMA fiber, including (**b**) carbon, (**c**) nitrogen, (**d**) sulfur, and (**e**) oxygen.

Figure S3 shows EDS elemental mappings of carbon (C), nitrogen (N), sulfur (S), and oxygen (O) distribution in the PANI/P3TI/PMMA fiber. It presented a homogenous element distribution in the blending fibers.

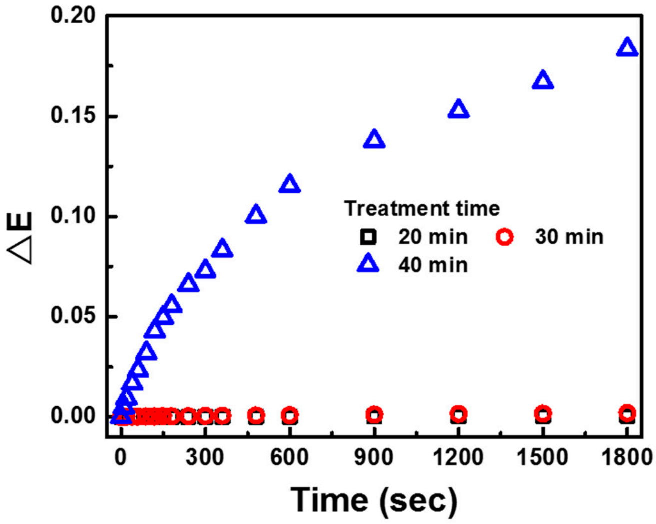

Figure S4. Extinction change at 620 nm of PANI/P3TI/PMMA fibers with various UV/ozone treatment time when exposed to 75% relative humidity.
